# Preliminary research on total nitrogen content prediction of sandalwood using the error-in-variable models based on digital image processing

**DOI:** 10.1371/journal.pone.0202649

**Published:** 2018-08-21

**Authors:** Zhulin Chen, Xuefeng Wang, Huaijing Wang

**Affiliations:** Research Institute of Forest Resource Information Techniques, Chinese Academy of Forestry, Beijing, China; Mar Ephraem College of Engineering & Technology, INDIA

## Abstract

This paper presents a method for predicting the total nitrogen content in sandalwood using digital image processing. The goal of this study is to provide a real-time, efficient, and highly automated nutritional diagnosis system for producers by analyzing images obtained in forests. Using images acquired from field servers, which were installed in six forest farms of different cities located in northern Hainan Province, we propose a new segmentation algorithm and define a new indicator named “growth status" (*GS*), which includes two varieties: *GS*_*MER*_ (the ratio of sandalwood pixels to the minimum enclosing rectangle pixels) and *GS*_*MCC*_ (the ratio of sandalwood pixels to minimum circumscribed circle pixels). We used the error-in-variable model by considering the errors that exist in independent variables. After comparison and analysis, the obtained results show that (1) The b and L channels in the Lab color system have complementary advantages. By combining this system with the Otsu method, median filtering and a morphological operation, sandalwood can be separated from the background. (2) The fitting degree of the models improves after adding the *GS* indicator and shows that *GS*_*MCC*_ performs better than *GS*_*MER*_. (3) After using the error-in-variable model to estimate the parameters, the accuracy and precision of the model improved compared to the results obtained using the least squares method. The optimal model for predicting the total nitrogen content is $y=237.374e^{-(4.471\frac{L}{L^{\prime }}+11.927\frac{a}{a\prime }+2.782\frac{b}{b\prime })}+26.248GS_{MCC}-4.274$. This study demonstrates the use of Internet of Things technology in forestry and provides guidance for the nutritional diagnosis of the important sandalwood tree species.

## Introduction

*Santalum album* L. is one of the tree species most widely used in perfume, medicine and advanced craft engraving. Almost all the advantages and benefits of sandalwood stem from the oil extracted from its core material [1], which is widely used in phytochemistry, pharmacology and other applications [2]. Due to the extensive demand for this oil in economic centers, sandalwood trees have been widely planted in southern China. However, sandalwood is difficult to nurture because it is sensitive to nitrogen, phosphorus, potassium and H_2_O levels. The nitrogen supply takes precedence over the others because nutrient deficiency or excess both affect heartwood growth; therefore, producers require a real-time and accurate nitrogen diagnostic method.

In plants, an insufficient nitrogen supply results in a smaller leaf area and a reduction in leaf photosynthesis, chlorophyll content and biomass production, leading to yield and quality losses [3,4]. Additionally, the excessive use of nitrogen-containing fertilizers not only increases production cost but also environmental pollution. However, agriculture and forestry fertilization currently depend on the experience of producers who are willing to apply nitrogen fertilizer in large amounts to ensure high yields over a range of environmental conditions.

The chemical composition of the soil can be used to identify the health of plants. The measures typically used assess the total nitrogen, effective nitrogen and inorganic nitrogen content (including nitric and ammonia nitrogen) to identify the amount of topdressing [5]. However, these methods are time-consuming and expensive [6,7,8], and there is no obvious relationship between the content of the nitrogen in the soil and in the crop. Plant sap analysis can address these disadvantages and has been successfully applied in the United Kingdom, Germany, Australia and the United States [9]; however, this method is destructive to plants and cannot provide rapid and automatic diagnostics.

Nondestructive testing technologies, including fertilizer windows, leaf color charts, chlorophyll meter reading (SPAD value), reflectance spectroscopy, hyperspectral remote-sensing technology and digital image-processing techniques, have developed rapidly in the past 20 years. However, each approach has shortcomings. For example, fertilizer windows cannot determine the specific application amount and also requires chemical analysis [10]. The leaf color chart method has difficulty determining the causes of color changes and is influenced by plant density and variety [11]. SPAD is used more widely for comparisons, but its measured leaf area is very limited [12], and when plant nitrogen content is close to or higher than the optimal value, the SPAD value cannot accurately characterize the chlorophyll content [13]. Reflectance spectroscopy has recently become highly popular in precision agriculture, but the canopy area measured by this method is relatively small and not representative of the actual value. Hyperspectral remote sensing technology is affected by the solar altitude angle, wind speed and soil humidity, and the required equipment is too expensive for many producers. Nevertheless, despite these disadvantages, hyperspectral remote sensing is likely to become an essential tool for large-scale nutritional diagnosis in the future.

Nutritional diagnosis based on digital image-processing technology is not only convenient and fast but also affordable because it does not require expensive data processing technology. Consequently, this method has been widely used in recent years. Researchers have analyzed leaf images using digital image-processing technology to provide support for diagnosing the nutritional status of crops [14]. Leaf color has been recognized as one of the most sensitive indicators of nutrient deficiencies [15], and nitrogen is directly related to leaf color because it is a key component of the chlorophyll molecule. Previously, researchers found a relationship between nitrogen content and leaf color that appears in images. Blinn *et al*. [15] used aerial photography to assess the need for fertilizers in loblolly pine plantations. Scharf *et al*. [16] established a linear model between the G/B value of the corn canopy and the minimum nitrogen application. After more than 30 years of development, nondestructive techniques for assessing nitrogen using digital images have been applied to rice (*Oryza glaberrima* L.), wheat (*Triticum aestivum* L.), maize (*Zea mays* L.), cotton (*Gossypium spp*.) and some other vegetables. Lee *et al*. [17] extracted the canopy coverage and ten types of color indicators for rice; they reported that those indicators can accurately match the differences caused by varieties and gradients. To estimate the required nitrogen fertilizer amount, an Android-based rice leaf color analyzer was proposed by Intaravanne *et al*. [18]; the key idea in this approach is to simultaneously capture and process the two-dimensional (2-D) color image data from rice leaves and their surroundings. In an experiment with wheat, Baresel *et al*. [19] used both image analysis and chlorophyll measurements to perform nondestructive detection. The results showed that chlorophyll measurements cannot reflect biomass, while image analysis can reflect both biomass and leaf nitrogen content. The development of color models, devices and artificial intelligence technologies have made image processing more convenient, and many new methods have been proposed. Romualdo *et al*. [20] used artificial vision techniques and digital image-processing to perform nitrogen nutritional-status diagnostics on maize; this approach can identify nutrient deficiencies at various stages of plant development, especially in the early growth stages. Confalonieri *et al*. [21] estimated leaf and plant nitrogen content using an 18%-gray dark green color index (DGCI) method. Compared to the DGCI and the corrected DGCI, the new method is considerably more stable with regard to both trueness and precision. Zhou *et al*. [22,23] used a ratio of vegetation index/leaf-area index called the RVI/LAI-reference curve to guide nitrogen fertigation and used image processing technology to estimate shoot nitrogen concentrations as well as the dry matter of potatoes. The results showed that normalized VI by ground cover was the best predictor for nitrogen estimation.

In China, studies on this technology started relatively later than in other countries. In 2007, Wang *et al*. [24] found a significant relationship between the color index of the plant canopy and nitrogen rate, yield, total nitrogen, plant nitrate concentration and biomass. Shortly afterward, image processing began to be widely applied for nutritional diagnoses. Domestic researchers have combined digital image-processing technology with plant soil testing and established corresponding recommended fertilization technology systems [25,26,27]. Zhang *et al*. [28] extracted leaf color characteristics using digital image-processing technology; their comprehensive evaluation showed that the quadratic polynomial established by G/(R-B) could obtain good prediction results: the coefficient of determination was 81.04%. Li *et al*. [29] found that the correlation index between various color factors (G, NRI, NGI, NBI, G/R and G/B) and nutrition parameters varies substantially as growth stages change, and the normalized red index (NRI) shows the optimal fit. Jia *et al*. [30] extracted green and red values from digital images of cotton, used them to calculate canopy cover and then calibrated the models to describe the relationship between the canopy cover and the aboveground total nitrogen content, biomass and LAI. Mao *et al*. [31] used a combination of spectroscopy and computer vision to conduct nondestructive nitrogen detection in lettuce. The computer vision approach extracted 11 plant features from images, including morphological, color and textural features, which improved the results.

Although nondestructive assessment of nitrogen from digital images has been widely applied in agriculture, it has been rarely used in forestry. Moreover, different crops have different image indicators that reflect their nutritional status, and the methods used in crops and fruits may not be suitable for trees. Sandalwood is economically valuable, and a large market demand exists, but due to excessive deforestation and ecological destruction, global sandalwood resources have declined sharply. China possesses no natural sandalwood resources, and because of restrictions in regions and financial conditions, the planting and cultivation of sandalwood in China is still in its infancy. Sandalwood generally requires 20–30 years to mature into useful timber, but that time can be reduced to 15 years through scientific management. To speed up sandalwood growth, the operators require real-time diagnosis techniques and reasonable fertilization. Therefore, nondestructive nutritional diagnosis based on digital image-processing technology provides a new approach to developing China’s sandalwood industry.

At present, numerous studies are focused on finding the image indicators that represent the nutrient content; however, these studies often ignore image segmentation effects, which could influence the optimization process. Regardless of the segmentation algorithm used, error always exists in color indicators: when defined as independent variables, color indicators increase system error because the independent variables in the model do not represent the true values. Simultaneously, nitrogen deficiency also affects plant growth status and can reveal whether a plant is deficient in nitrogen. However, growth status has not been studied as a model indicator during model construction in previous research. Therefore, the goals of this study are threefold: i) to propose an image segmentation algorithm suitable for field images; ii) to use growth status as a model indicator to predict the total nitrogen content of sandalwood; and iii) to use the error-in-variable model to estimate the parameters to produce a more reliable result.

## Materials and methods

### Research area survey and data acquisition

**Fig 1:**
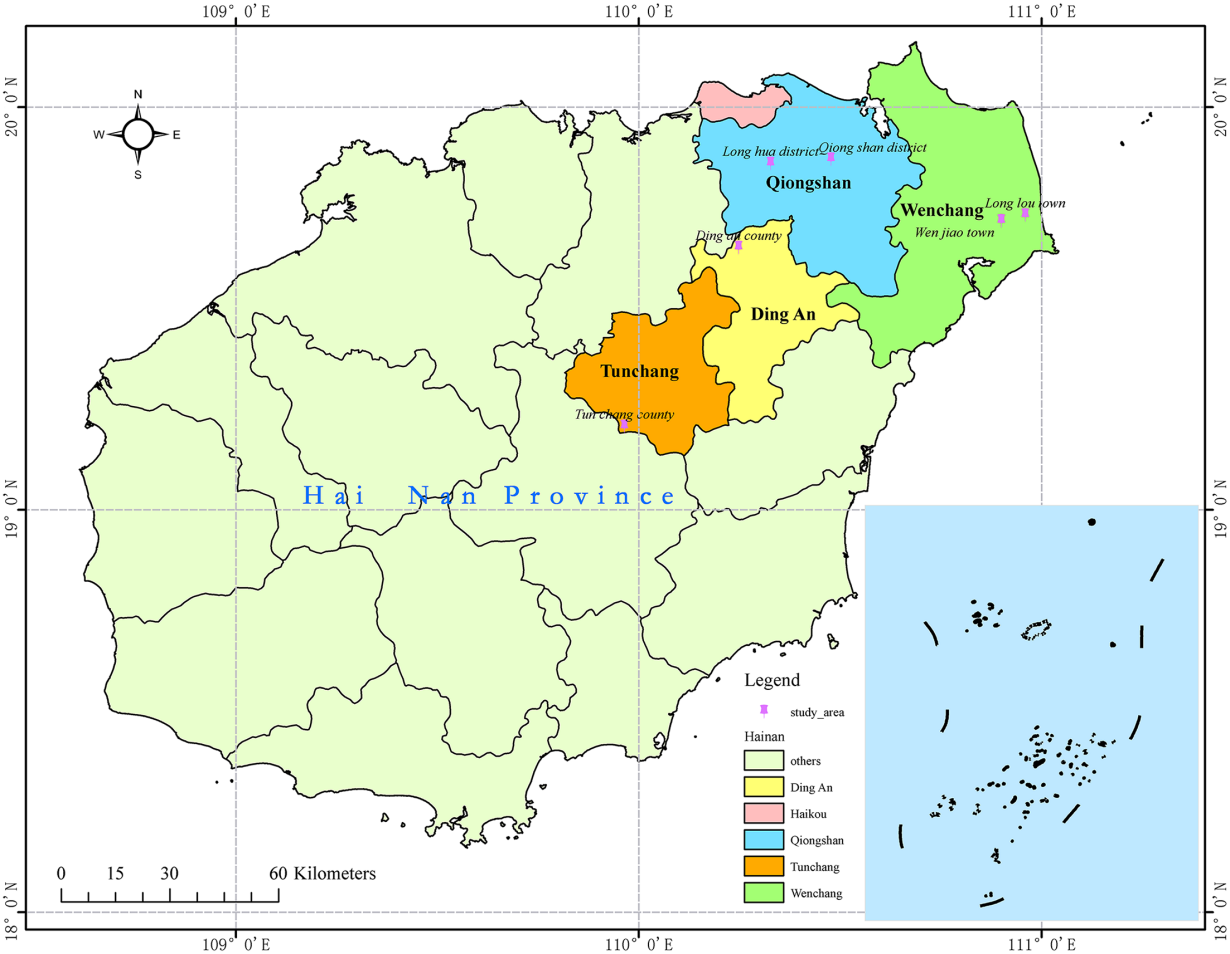
Distribution of sandalwood samples in northern Hainan Province.

The data used in this paper were collected from different forestry centers in northern Hainan Province containing planted sandalwood trees. To enhance the data representation, the samples were collected from cities that differ in location and soil type. We chose six study areas located in four different cities as shown in Fig 1. The soil types of the study areas are shown in Table 1. Supplementary soil sample descriptions can be found in S1 Table.

**Table 1:** Soil types among the different study areas.

Study area	Soil type	Geographical location
*Longhua District*	Yellow-brown earth	19°51′25″N~19°53′43″N, 110°19′50″E~110°21′26″E
*Qiongshan District*	Red earth	19°52′17″N~19°54′05″N, 111°28′38″E~111°29′56″E
*Long Lou Town*	White sandy loam	19°43′58″N~19°44′58″N, 110°57′34″E~110°57′50″E
*Wenjiao Town*	Red sandy loam	19°42′05″N~19°43′56″N, 110°52′59″E~111°54′01″E
*Ding’an County Seat*	Dark-brown earth	19°39′08″N~19°41′56″N, 110°14′51″E~110°16′05″E
*Tunchang County Seat*	Latosol	19°11′28″N~19°13′31″N, 109°57′52″E~109°59′18″E

In agricultural, crops, plants are relatively short; thus, canopy images can easily be acquired for use in segmentation [32,33]. However, for forestry, an unmanned aerial vehicle (UAV) is necessary to perform low-altitude remote sensing to obtain tree canopy images. Not only does this requirement increase costs but it also has unsolved problems such as shadows caused by leaves, which affect the reflection information of the canopy when performed on a sunny day [34]. In this research, we used a field server as ground remote-sensing equipment to monitor the sandalwood. A field server is a real-time monitoring device consisting of a CCD camera, air temperature, humidity and soil temperature sensors and a wireless local area network module. From 6:00 to 18:00, the cameras captured images every hour and transmitted them to the server in real time. To reduce the optical illumination effect, we selected images of 1024×768 pixels taken between 12:00 and 14:00.

The sandalwood saplings studied in this research were all started from seedlings. The seedlings were grown in nurseries to the 8–10-leaf stage and then transplanted into the forest farms. After 4 years, 12 saplings were chosen from each farm for the experiment, and field servers were placed near them. Nitrogen as urea was applied at 4 rates: 0 (N0), 80 (N1), 160 (N2) and 240 (N3) kg/ha. Each rate was applied randomly to groups of 3 saplings at each study area.

### Sampling and testing

**Fig 2:**
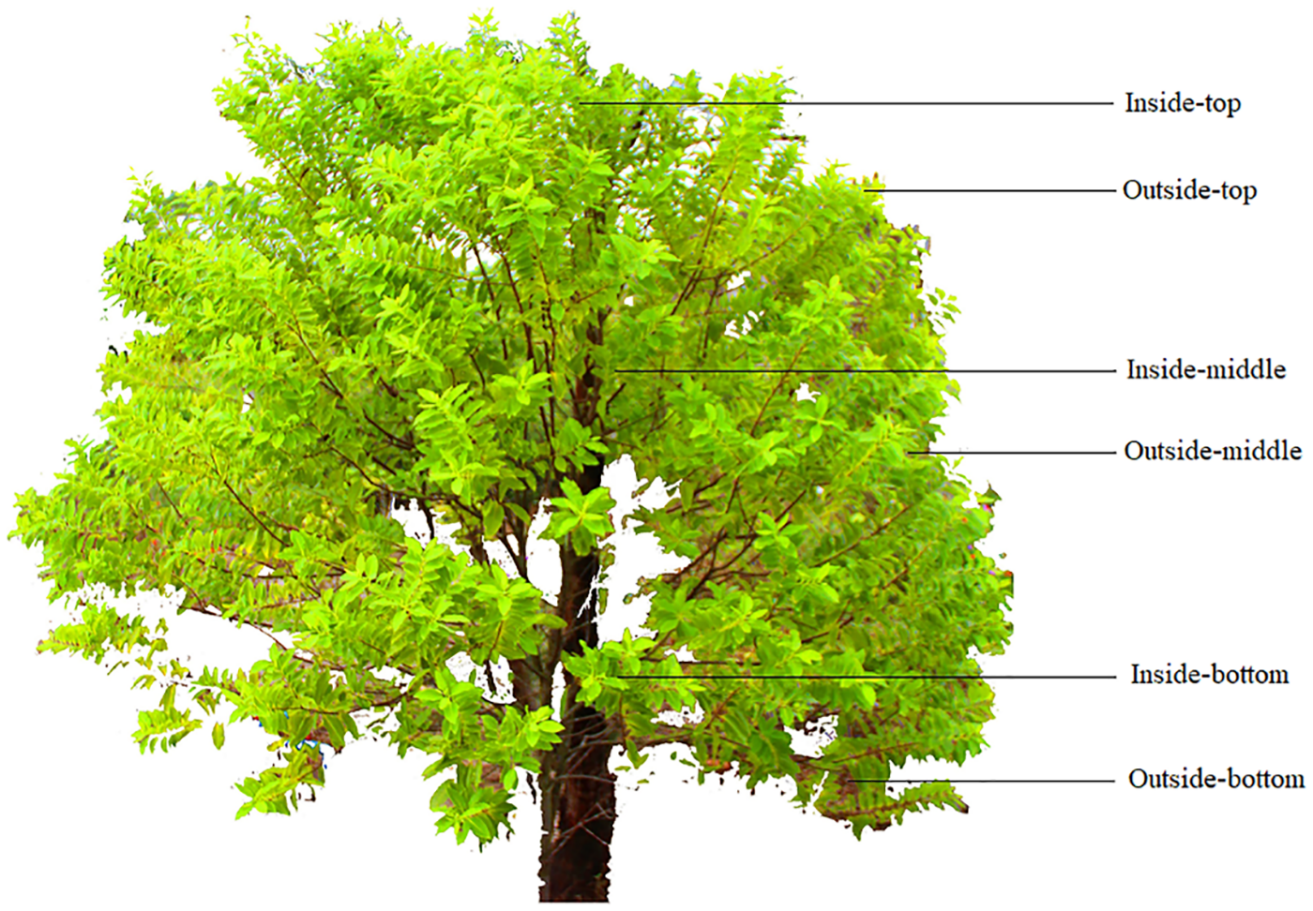
Sampling method.

Leaf sampling was required after the image data were collected. According to the transmission mechanism of nitrogen, the nitrogen content in leaves varies with changes in the growth position. To ensure that the data were representative, the sampling test was divided into 6 levels (inside-top, outside-top, inside-middle, outside-middle, inside-bottom and outside-bottom) as shown in Fig 2. Several leaves—both old and new—were collected at each level. The collected leaves were dried at 85 °C until the weight remained constant, ground through a 20-mesh screen and analyzed for total nitrogen via Micro-Dumas combustion by the soil testing and plant analysis laboratory at the Chinese Academy of Tropical Agricultural Sciences.

### Data analysis

Image data processing was implemented using the MATLAB R2012a platform, and data analysis was performed using SPSS Statistics 21.0 and the R Language. RGB is the most commonly used color space for storing and displaying color images, but it is susceptible to illumination and shadow effects. In contrast, the HSI color space is relatively stable to changes in light intensity [35]. CIELAB is a device-independent color system based on physiological characteristics that can express a color range larger than the human eye can see. To analyze the correlations between color values and total nitrogen content, the single-channel mean values of the RGB, HSI and Lab color spaces were calculated after segmenting the sandalwood from the complex background.

The total nitrogen content not only affects leaf color but also significantly influences aboveground biomass. Therefore, in addition to the color factors, a new indicator is proposed in this paper, “growth status” (*GS*), represented by *GS*_*MER*_ and *GS*_*MCC*_ which are discussed separately. The definition of growth status is as follows.

After segmentation, the minimum enclosing rectangle (*MER*_*p*_) and minimum circumscribed circle (*MCC*_*p*_) are searched. Then, the number of sandalwood pixels *S*_*p*_ in *MER*_*p*_ and *MCC*_*p*_ are calculated. *GS*_*MER*_ and *GS*_*MCC*_ are the ratios of *S*_*p*_ to *MER*_*p*_ and *MCC*_*p*_, respectively:

$$GS_{MER}=\frac{S_{p}}{MER_{p}},\;GS_{MCC}=\frac{S_{p}}{MCC_{p}}$$

### Error-in-variable model

In the typical regression model, independent variables are regarded as true values, while the dependent variables have measurement errors. The errors of the variables have various sources such as sampling error, observation error, and so on. However, the independent variables may also contain errors from different aspects. In all models, it is assumed that these errors have a random distribution. We call those random errors the error-in-variable. However, the typical regression model estimation method is not appropriate when errors exist in both the dependent and independent variables. In particular, when the measurement error in the independent variables are relatively large, the results calculated using the conventional method will produce obvious systematic errors. Therefore, we must use the error-in-variable model to estimate the parameters [36,37].

The error-in-variable model is a parameter estimation algorithm as described in [38]:

$$\{\begin{array}{l} f(y_{i},x_{i},c)=0\\ Y_{i}=y_{i}+e_{i},i=1,\ldots n\\ E(e_{i})=0,Var(e_{i})=\Sigma \end{array},$$

where *f* = (*f*_1_, *f*_2_,…,*f*_*m*_)’ is a known vector-valued function in the m dimension; *Y*_*i*_ in the 1×p dimension is the true value of *y*_*i*_—the observation value; *e*_*i*_ is the error between *Y*_*i*_ and *y*_*i*_; *x*_*i*_ in the 1×q dimension is the observation value with no error; ∑ is the positive definite matrix, which can be known or unknown; and c is a parameter sized in the k×1 dimension. In general, *p* ≥ *m*. When *f* is a bilinear function of (*y*_*i*_, *x*_*i*_) and c, the model is called a linear error-in-variable model; otherwise, it is a nonlinear error-in-variable model.

Due to the influence of light and measurement, errors always exist in both independent variables and dependent variables in this research. Thus, the use of the error-in-variable model methods can ameliorate this problem and improve the prediction ability of the model.

### Model evaluation

This study selected 48 groups of data randomly for modeling and then evaluated the model with the remaining 24 groups of samples. The images used for modeling and validation are shown in S1 and S2 Figs. The adopted statistical parameters—coefficient of determination (*R*^2^), residual mean value ($\bar{e}$), residual variance value (*δ*^2^) and mean square error (*MSE*)—represent the differences between the measured and predicted values [39]. Eqs (1) and (2) were used in modeling to show the fitting degree, and Eqs (1)–(4) were used during validation to show the precision of the models:

$$R^{2}=1-\frac{\sum_{i=1}^{n}\left(y_{i}-y_{i}^{\prime }\right)}{\sum_{i=1}^{n}\left(y_{i}-{\bar{y}}_{i}\right)}$$ (1)

$$\bar{e}=\frac{1}{n}\sum_{i=1}^{n}\left(y_{i}-y_{i}^{\prime }\right)$$ (2)

$$\delta^{2}=\frac{1}{n-1}\sum_{i=1}^{n}{\left(y_{i}-y_{i}^{\prime }\right)}^{2}$$ (3)

$$MSE=\sqrt{{\bar{e}}^{2}+\delta^{2}}$$ (4)

where *y*_*i*_, $y_{i}^{\prime }$, ${\bar{\text{y}}}_{i}$ and *n* are the observed values, predicted values, mean of the observed values and number of samples, respectively.

## Results and discussion

### Segmentation algorithm in a complex background

**Fig 3:**
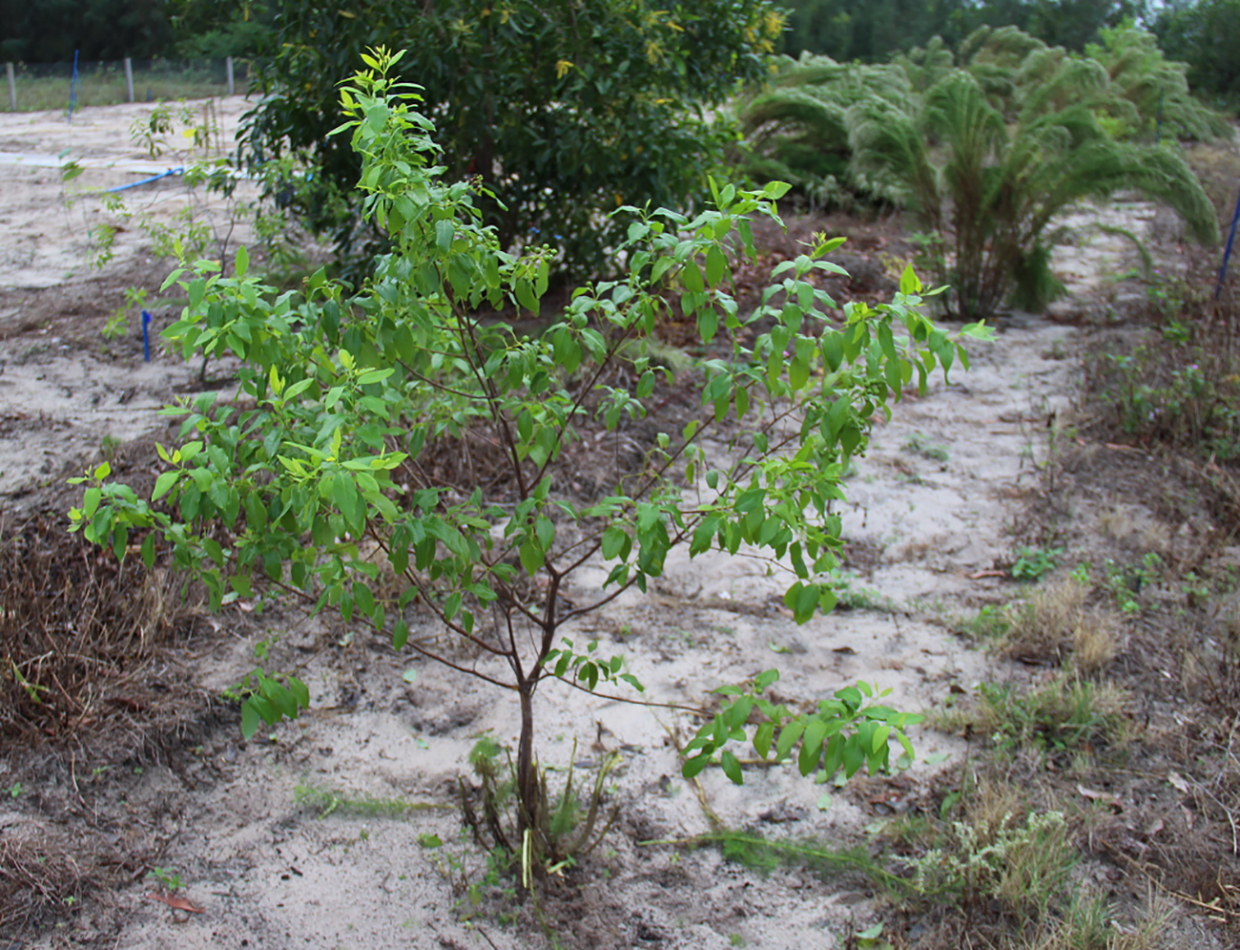
Original sandalwood image.

As shown in Fig 3, soil, weeds and other green plants exist in image backgrounds and cause difficulties during image segmentation. Therefore, to ensure the quality of subsequent work, it was necessary to propose an accurate image segmentation algorithm.

The CIELAB color model is the most complete color system; it can describe all the colors visible to the human eye. It has a large color space compared with the RGB system and is more robust to illumination changes; thus, it is suitable for analyzing images acquired in the field. In the Lab color system, L represents brightness, which ranges from 0–100, and the "a" and "b" represent different color channels ranging from -128 to 127.

**Fig 4:**
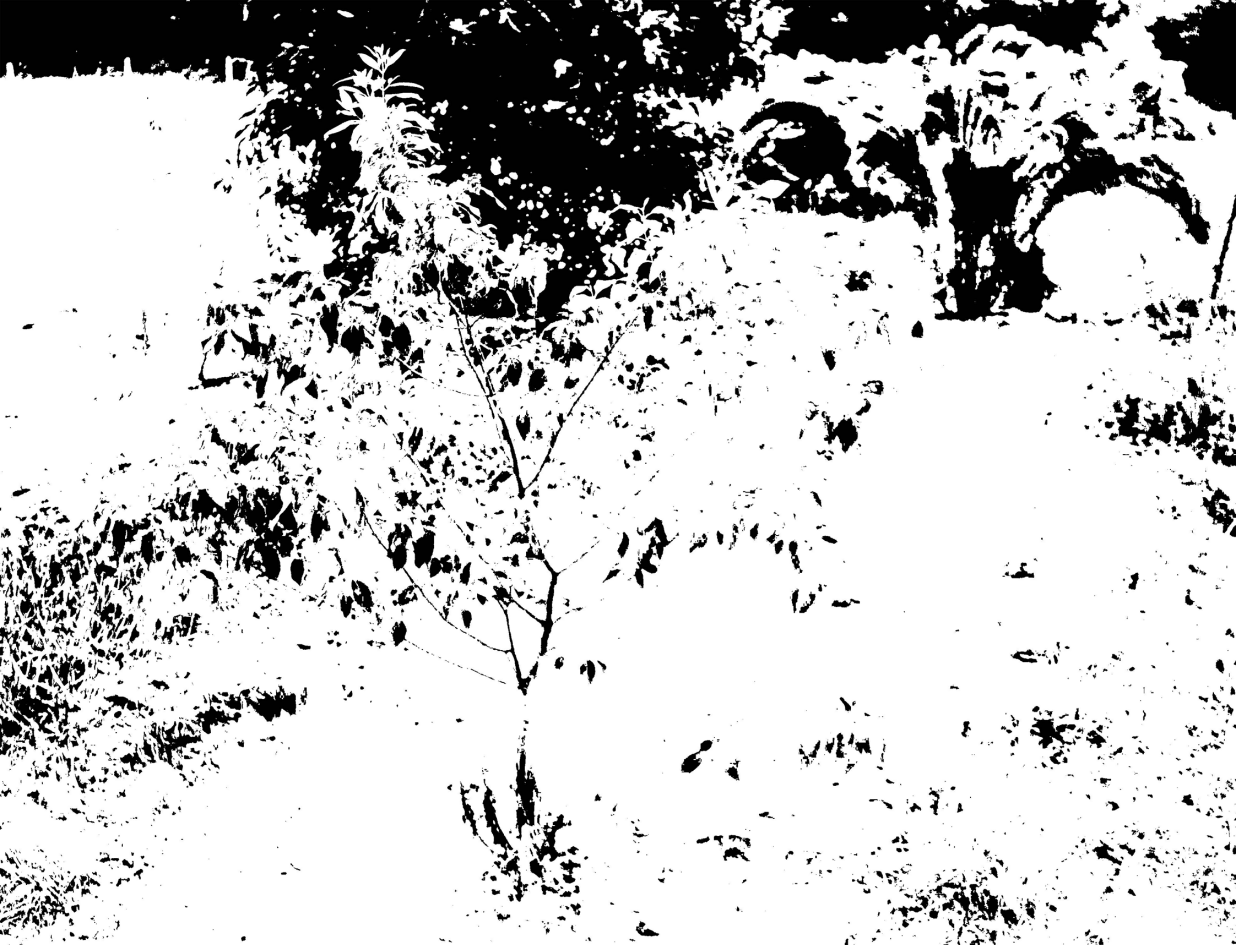
Segmentation result of Lab image using the Otsu method.

**Fig 5:**
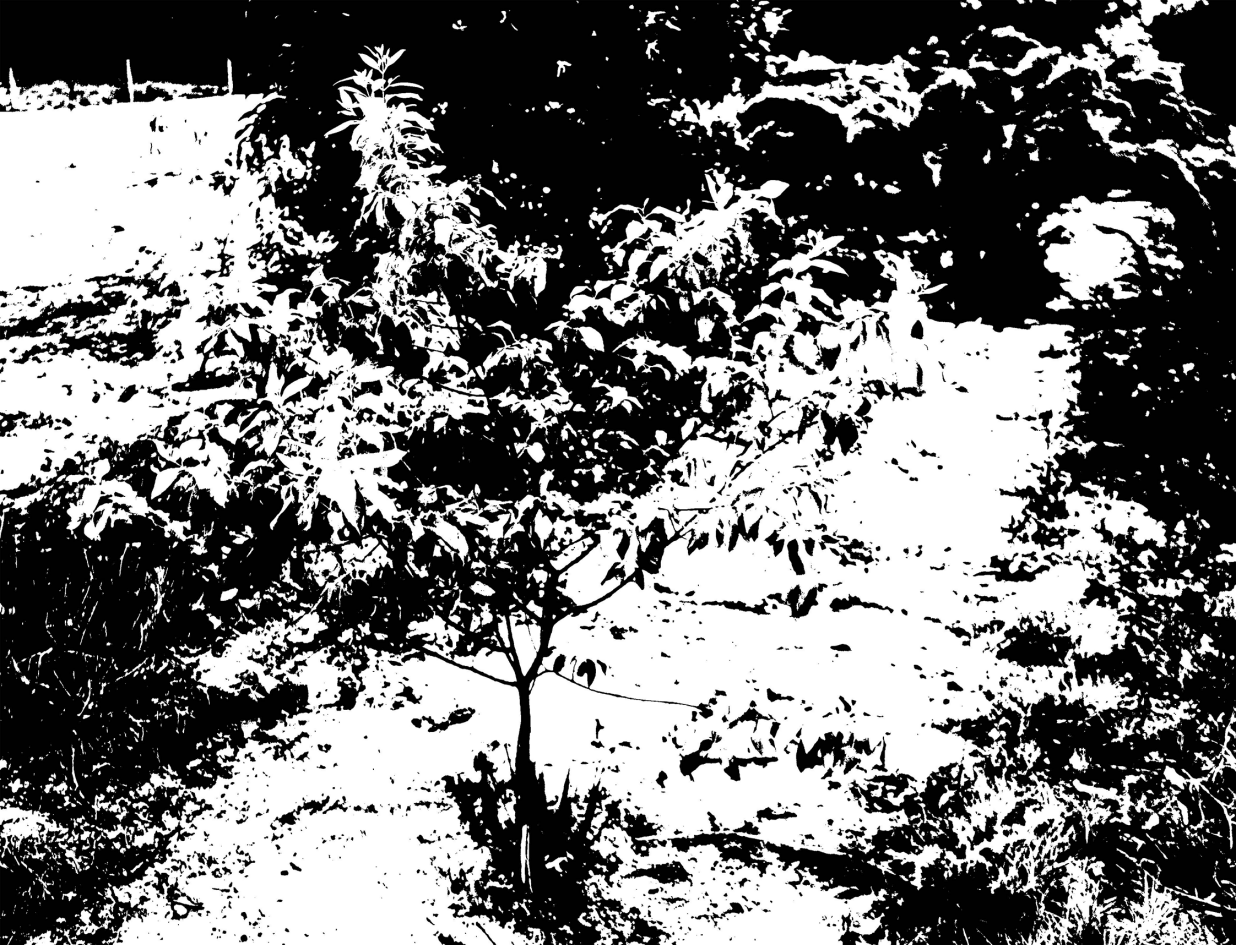
Segmentation result of the L channel image using the Otsu method.

**Fig 6:**
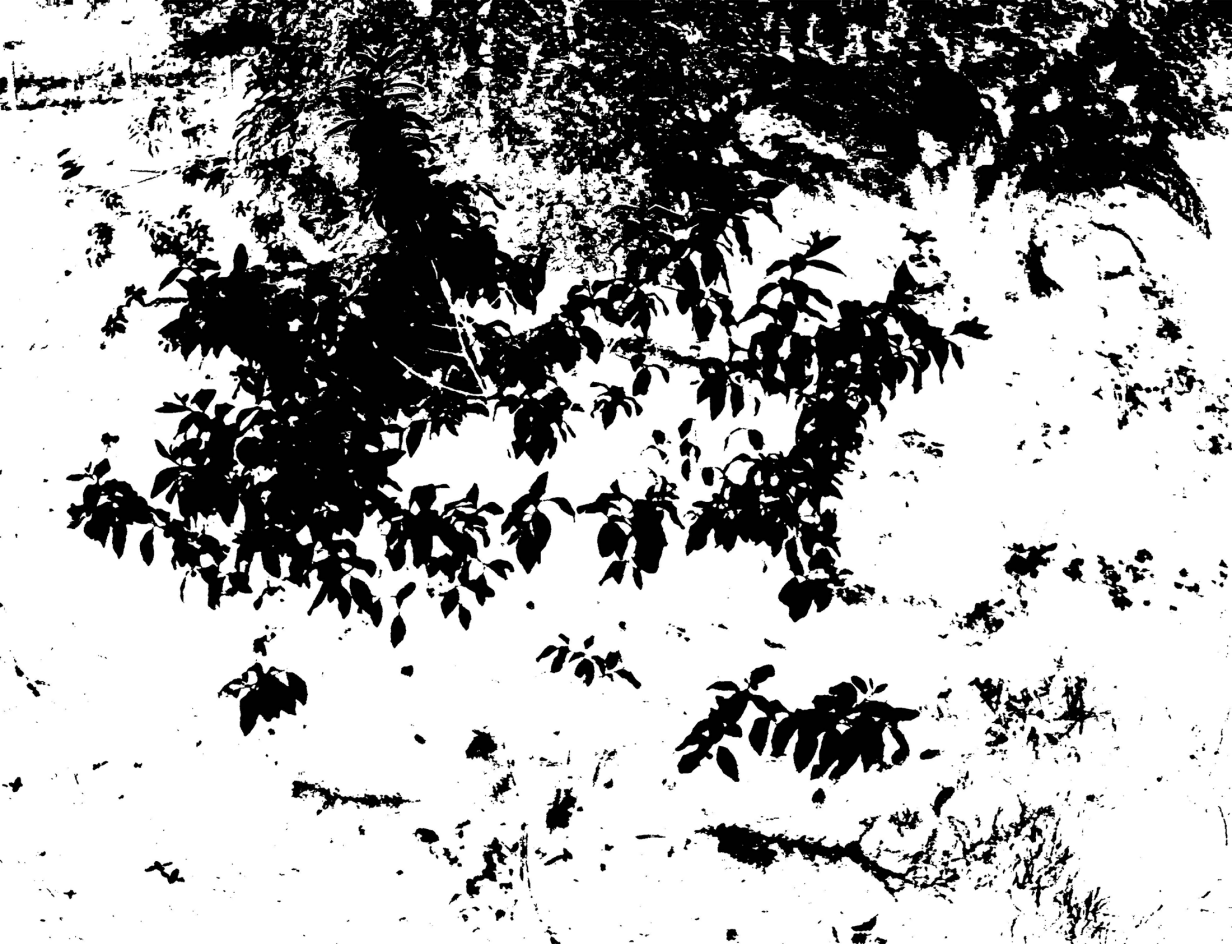
Segmentation result of the a channel image using the Otsu method.

**Fig 7:**
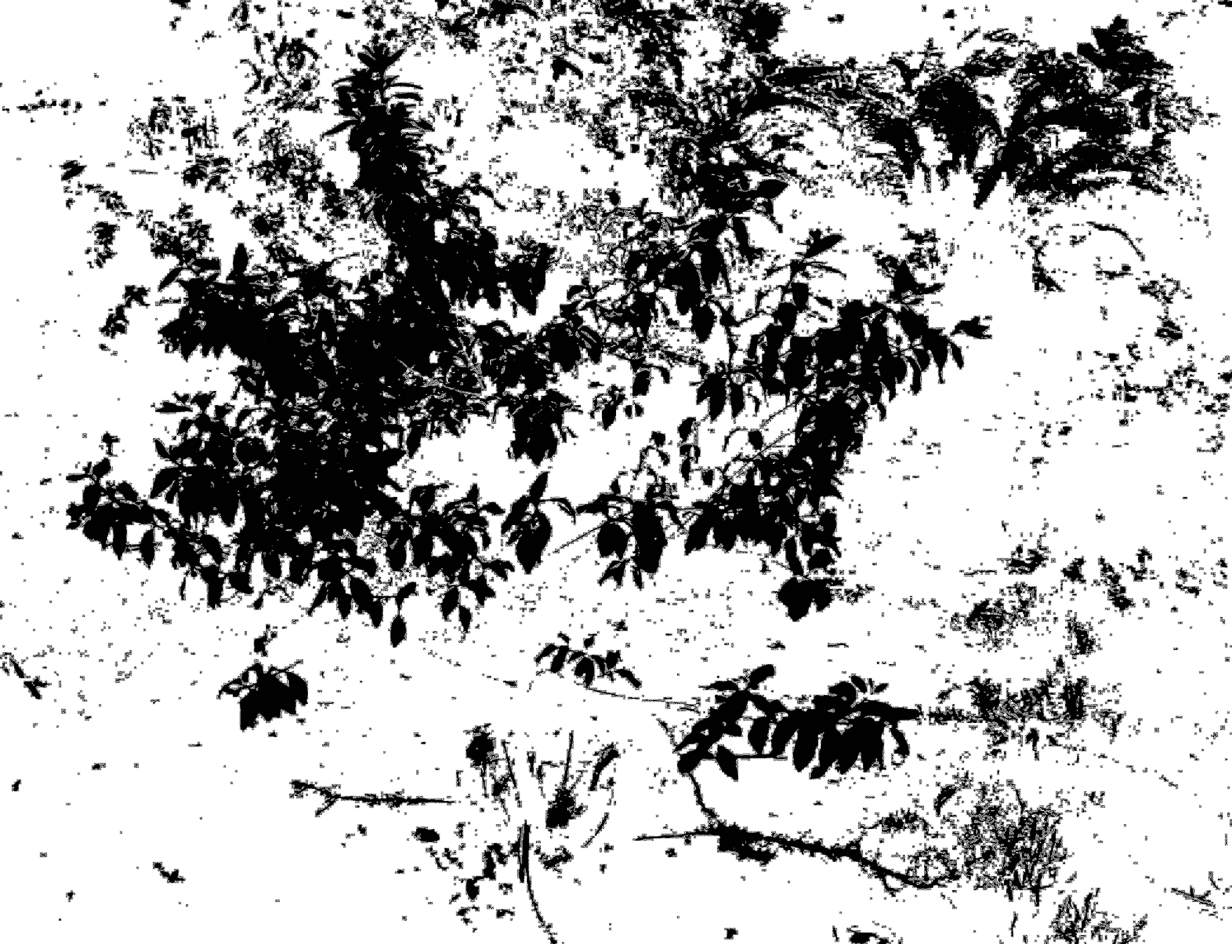
Segmentation result of the b channel image using the Otsu method.

Due to the complexity of the field images, the traditional RGB color system is unsuitable for obtaining an accurate segmentation result; using the Lab color space eliminates this problem. In this study, we transformed RGB into the Lab system and then extracted the L, a and b channels and conducted the Otsu method to obtain a binary image. Through a large number of experiments, we found that the Lab color image is suitable for dividing sandalwood and soil into classes while the a and b channels are suitable for separating sandalwood leaves from soil and other tree species. Of these, the b channel obtained a better result in comparisons. Nevertheless, some background pixels still remained when using only the a or b channel. Through experimentation, we found that the L channel can fill in the gaps, allowing sandalwood to be separated from other plants through brightness, which eliminated most of the residual pixels. Thus, we chose the b and L channels to conduct the image segmentation. The results are shown in Figs 4 to 7.

The process of the segmentation algorithm is as follows.

**Fig 8:**
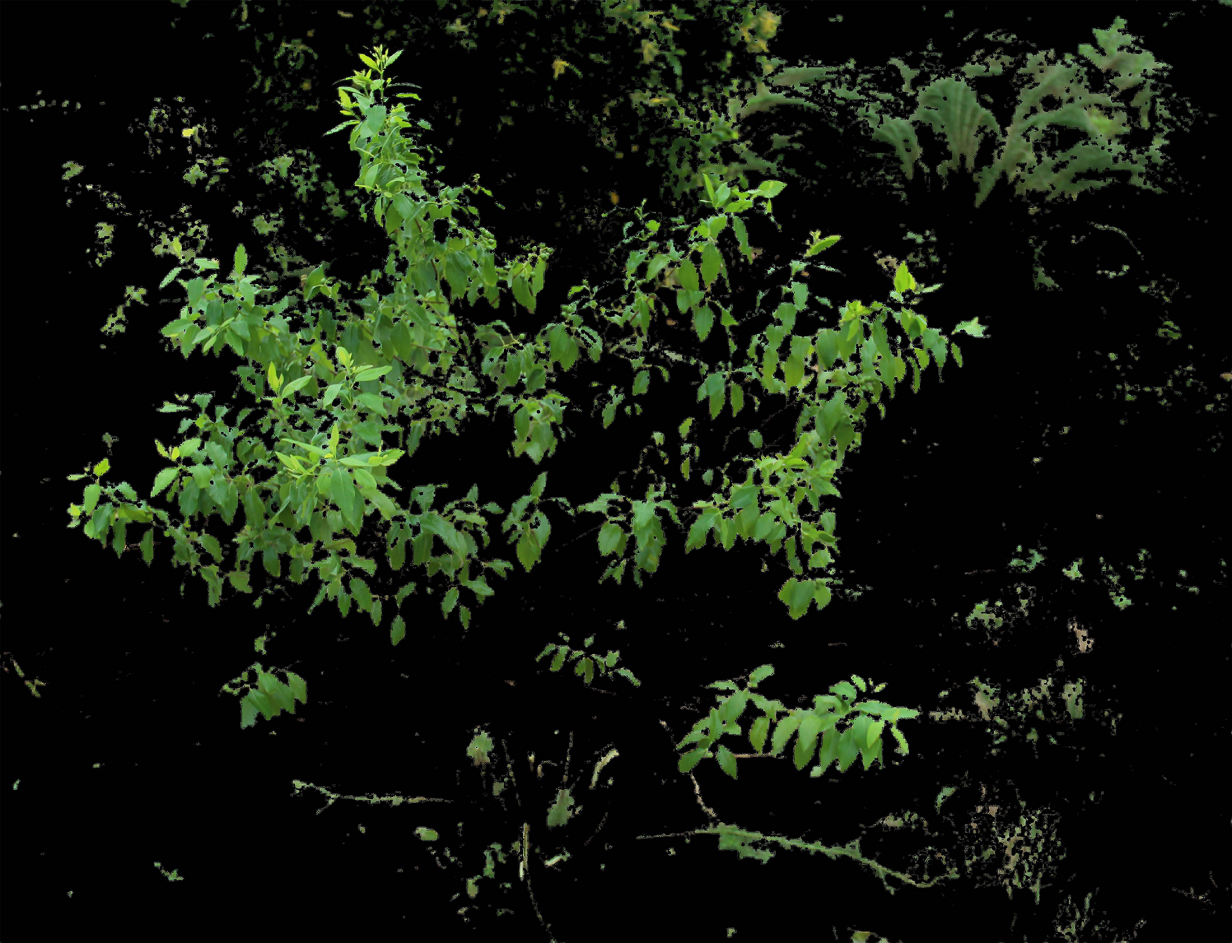
Segmentation result after median filter processing.

**Fig 9:**
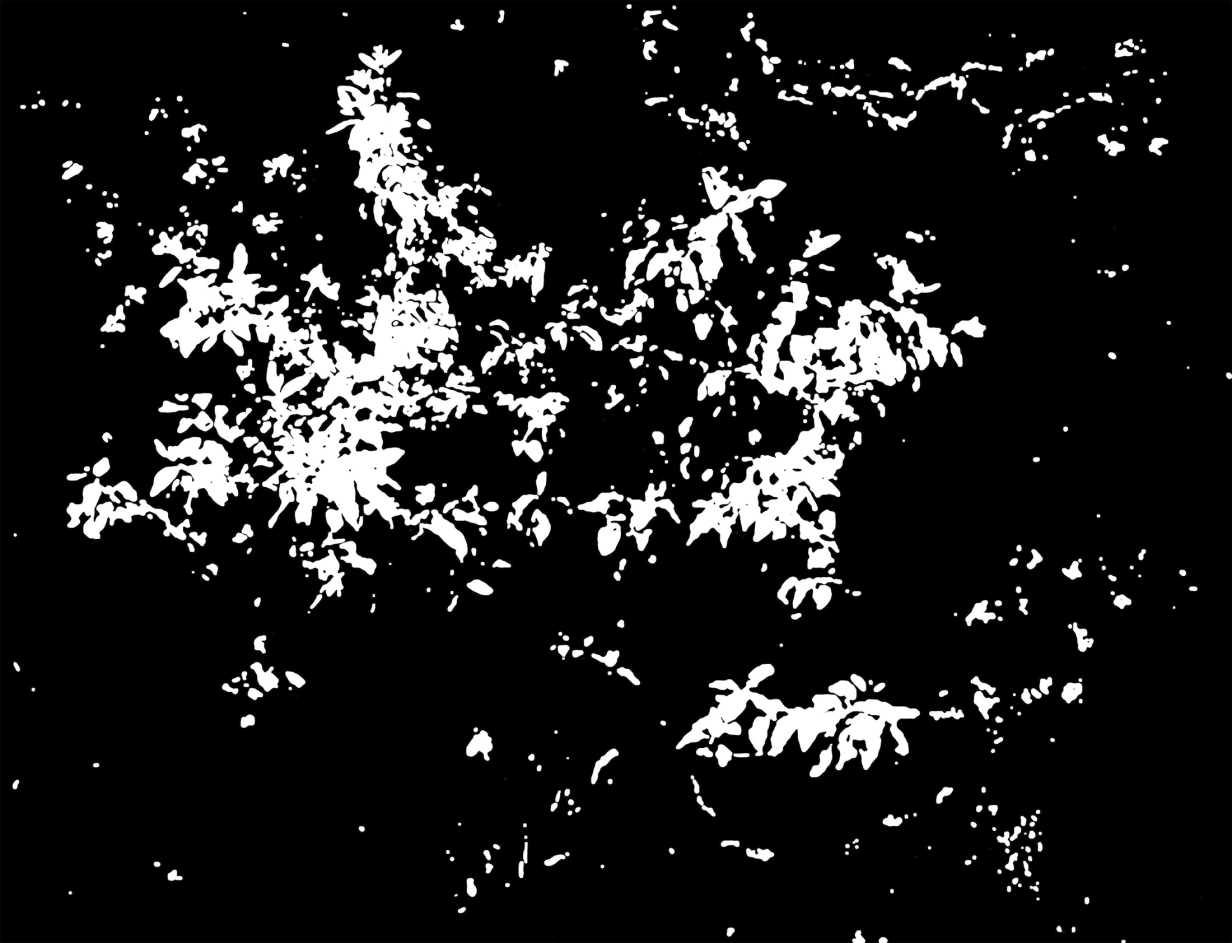
Threshold segmentation result.

**Fig 10:**
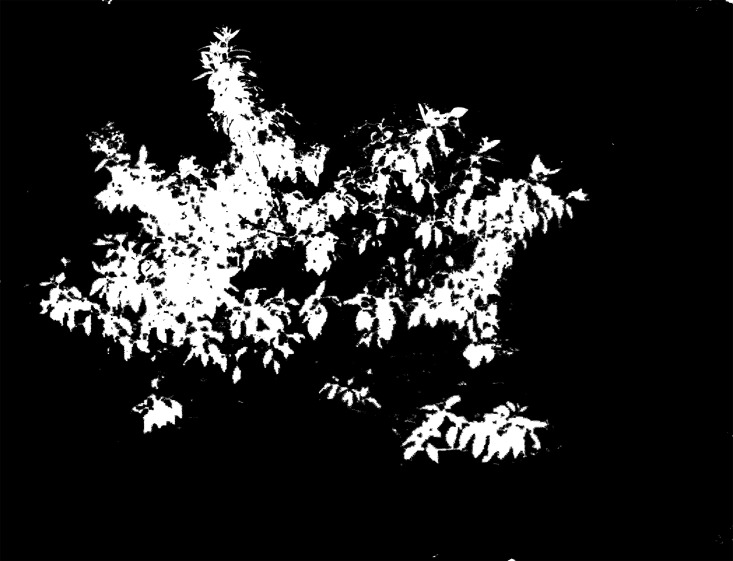
Morphological processing.

**Fig 11:**
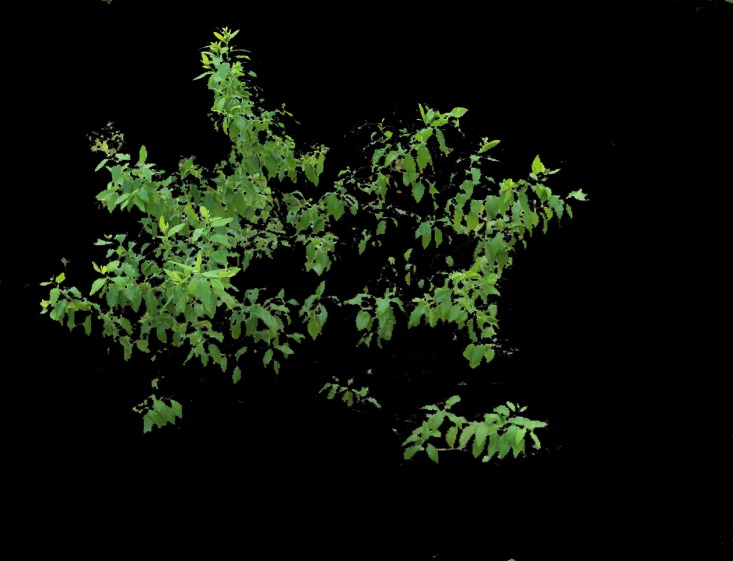
Final segmentation result.

1. Convert the RGB image into the Lab color space; then, extract the b and L channels.
2. Use the Otsu method to convert the b-channel and L-channel images into binary images. Record the thresholds as *T*_*L*_ and *T*_*b*_. The binary images are recorded as *I*_*L*_ and *I*_*b*_.
3. Perform median filter processing (7×7) on *I*_*b*_ and then multiply it by the original image. The processing result is recorded as *I*_*b*1_ (Fig 8).
4. Convert *I*_*b*1_ into the Lab color space and extract the L channel; then, use the threshold *T*_*L*_ to convert it to binary, and record the result as *I*_*b*2_ (Fig 9).
5. Perform median filter processing (7×7) on *I*_*b*2_ and then use the circular structure element with a radius of 5 to corrode twice and expand twice (Fig 10). Multiply it by the original image and record the final segmentation result as *I*_*b*3_ (Fig 11).

After numerous comparisons and analyses, the template and the structural elements used in the algorithm were found to be the best choice. As shown in Figs 8 to 11, image *I*_*b*_ eliminates the soil background and plants with a dark green color. The L-channel is used to separate sandalwood from other plants using brightness differences, eliminate scattered pixels through median filtering and morphological operations, and then, to produce the final segmentation results.

To verify the algorithm proposed in this paper, we compared it with the results obtained using both a support vector machine (SVM) method and the results of manual processing with Photoshop software. The results obtained by Photoshop, which are processed using the polygonal lasso tool, are equivalent to visual interpretation; therefore, we adopted these results as the ground truth. Seventy images containing both the modeling data and the validation data were tested. We used a color indicator to diagnose the total nitrogen; therefore, the evaluation criteria included not only the number of pixels but also the mean values of the RGB channels. All the images tested resulted in a pixel number error within 5% and a color error within 3%, which indicated that the segmentation algorithm was appropriate. As listed in Table 2, five images were chosen randomly to show the comparison results. The pixel number errors obtained through the SVM approach are relatively larger than those obtained by the method proposed in this paper. This discrepancy could cause deviations when finding the minimum enclosing rectangle and minimum circumscribed circle for segmenting sandalwood. In addition to the pixel error, the color value errors obtained by the two methods differed slightly; the proposed method achieved more stable results.

**Table 2:** Segmentation method evaluation proposed in this paper.

Number	Methods	Pixel number error (%)	R mean value	Error (%)	G mean value	Error (%)	B mean value	Error (%)
**(1)**	①	-	177.11	-	219.99	-	97.13	-
	②	2.85	175.32	1.01	222.83	1.29	99.76	2.71
	③	5.94	174.65	1.39	217.93	0.94	101.32	4.31
**(2)**	①	-	196.31	-	227.14	-	102.12	-
	②	3.94	198.37	1.05	225.93	0.53	104.93	2.75
	③	6.46	198.12	0.92	224.84	1.01	105.99	3.79
**(3)**	①	-	166.96	-	208.85	-	103.16	-
	②	3.37	167.38	0.25	209.71	0.41	101.33	1.78
	③	6.92	168.65	1.01	208.88	0.01	100.43	2.65
**(4)**	①	-	199.63	-	233.15	-	94.07	-
	②	3.09	201.39	0.88	235.22	0.88	92.48	1.69
	③	8.67	205.39	2.89	237.43	1.84	91.45	2.79
**(5)**	①	-	185.59	-	219.81	-	102.10	-
	②	4.53	183.73	1.00	216.28	1.61	104.73	2.58
	③	6.65	188.39	1.51	223.04	1.47	103.45	1.32

^①^ Represents the results obtained by Photoshop.

^②^ Represents the results obtained by the segmentation algorithm proposed in this paper.

^③^ Represents the results obtained by the SVM method.

### Parameter selection and model construction to determine the total nitrogen content in sandalwood

As shown in Table 3, significant correlations were observed among the total nitrogen content, color factors, and *GS* indicators. Except for the a and S channels, the other factors are both significantly correlated with total nitrogen content at the 0.01 level. The R, L and I channels have the strongest Pearson correlation values; thus, they were selected for combination in a mixed-color system and were compared with the other color systems when building the model.

**Table 3:** Results of significance tests and Pearson correlations between factors and nitrogen content (*m* = 48).

Dependent variable	Pearson	Dependent variable	Pearson
**R**	-0.875**	**L**	-0.815**
**G**	-0.775**	**a**	0.203
**B**	-0.671**	**b**	-0.734**
**H**	-0.543**	***GS***_***MCC***_	0.789**
**S**	-0.426*	***GS***_***MER***_	0.524**
**I**	-0.808**	**-**	-

** Indicates a significant difference at the 0.01 probability level (two-tailed).

* Indicates a significant difference at the 0.05 probability level (two-tailed).

**Fig 12:**
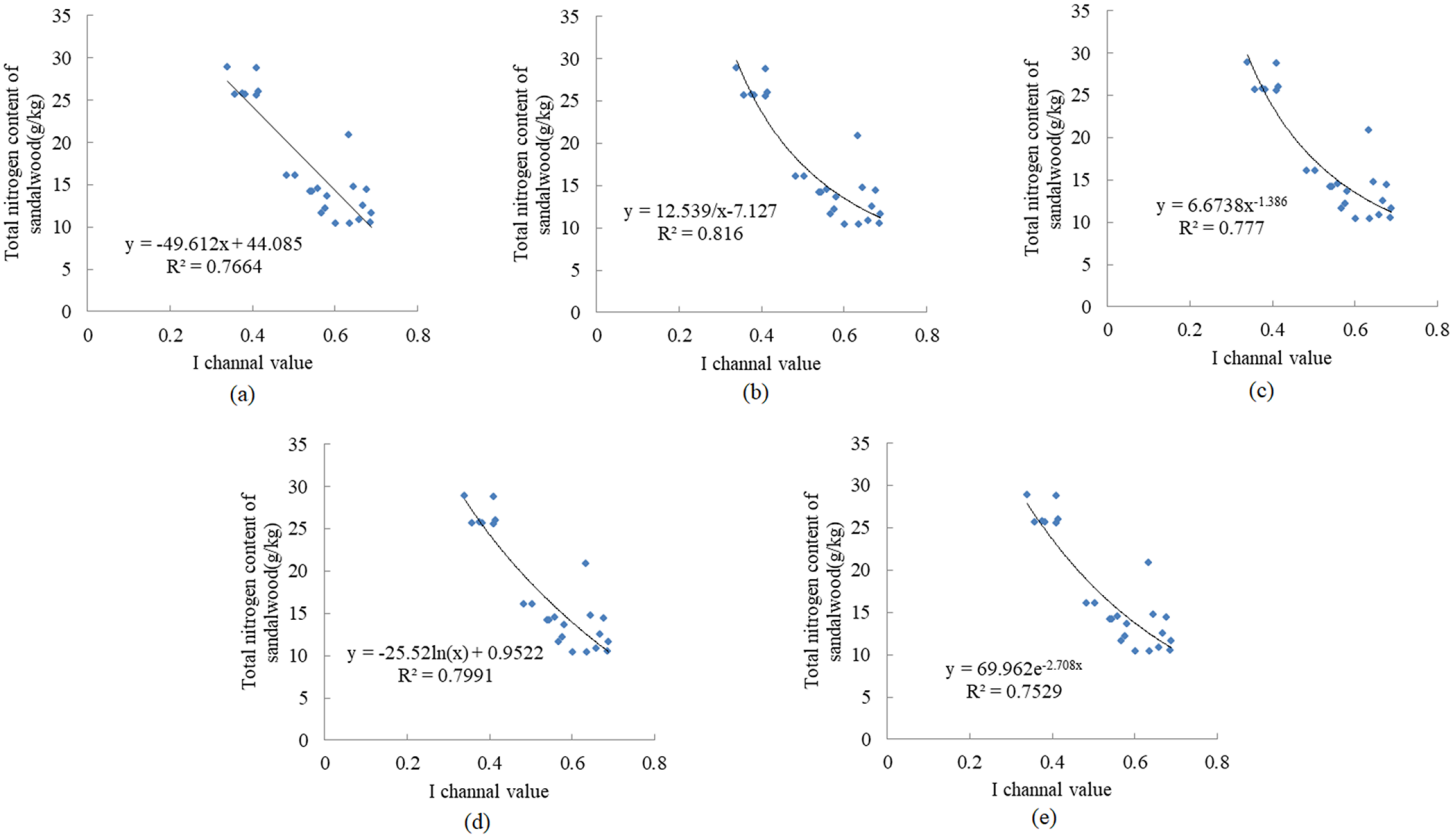
Relationship between I channel value and sandalwood total nitrogen content. (a) Linear function; (b) Reciprocal function; (c) Power function; (d) Logarithmic function; (e) Exponential function.

Color value and leaf total nitrogen content generally show a linear or nonlinear trend that can be expressed as linear, reciprocal, power, logarithmic or exponential functions (Table 4). Thus, those formulas are commonly used to predict nitrogen content, biomass and chlorophyll content [40–42]. For example, the relationship between the value of the I channel and total nitrogen of sandalwood is shown in Fig 12. All the obtained models satisfied the fitting results.

**Table 4:** Prediction models of total nitrogen content in sandalwood.

Model	Expression
**Linear Combination**	$y=a_{gs}+\sum_{i=1}^{3}a_{i}x_{i}$
**Reciprocal Combination**	$y=a_{gs}+\sum_{i=1}^{3}\frac{a_{i}}{x_{i}}$
**Power Function**	$y=a_{gs}+a_{0}\prod_{i=1}^{3}{\left|x_{i}\right|}^{a_{i}}$
**Logarithmic Function**	$y=a_{gs}+a_{0}\text{ln}\left|\sum_{i=1}^{3}a_{i}x_{i}\right|$
**Exponential Function**	$y=a_{gs}+a_{0}e^{\left(\sum_{i=1}^{3}a_{i}\frac{x_{i}}{x_{i}{}^{\prime }}\right)}$

In every expression, *a*_*gs*_ = *b*_0_ + *b*_1_*GS*_*MER*_ or *a*_*gs*_ = *b*_0_ + *b*_1_*GS*_*MCC*_, both of which are discussed in this paper. Here, *x*_1_, *x*_2_ and *x*_*3*_ are the mean values of the single channel in each color system. In the exponential functions, $x_{i}^{\prime }$ represents *x*_*i*max_ − *x*_*i*min_.

### Regression analysis of the total nitrogen content prediction model of sandalwood

Because the best version of the five models’ expressions for each color system is unknown, each model type was tested against 4 color systems. To select the best color system and *GS* indicator to estimate the total nitrogen of sandalwood, the linear, reciprocal, power, logarithmic and exponential models were selected for regression analysis, and 3 groups of data (color, color and *GS*_*MER*_, color and *GS*_*MCC*_) were set up to test the influence of the different *GS* indicators.

As shown in the ninth column of Table 5, the *R*^2^ values of each model range from 0.812 to 0.848, and the $\bar{e}$ values range from -0.424 to -0.234 without adding the *GS* factor. The differences between the color systems for any given model is small, and—except for the power function—the fit of the Lab system result is better than the others. With respect to $\bar{e}$, the Lab system obtained a better result in the linear, reciprocal, and power models, while the RGB system obtained a better result in the logarithmic and exponential models. After adding *GS*_*MER*_ and *GS*_*MCC*_, *R*^2^ increased to ranges of 0.841~0.885 and 0.863~0.913, respectively, and $\bar{e}$ was reduced to ranges of -0.402~-0.184 and -0.394~-0.158, respectively. This indicated that the fitting degree obtained when using the minimum circumscribed circle is better than that when using the minimum enclosing rectangle. The parameter in Table 6 lists the model coefficients after adding the *GS*_*MCC*_ indicator.

**Table 5:** Regression analysis results of the least square method using different indicators (*m* = 48).

Model	Color System	Coefficient						*R*^2^			$\bar{e}$		
		*a*_0_	*a*_1_	*a*_2_	*a*_3_	*b*_0_	*b*_1_	Color factor	Color factor+*GS*_*MER*_	Color factor+ *GS*_*MCC*_	Color factor	Color factor+*GS*_*MER*_	Color factor+ *GS*_*MCC*_
**Linear Combination**	RGB	-	0.031	-6.496	43.648	0.203	-0.418	0.824	0.841	0.878	-0.301	-0.298	-0.294
	HSI	-	-44.606	-10.707	52.013	0.405	-35.07	0.831	0.852	0.863	-0.289	-0.274	-0.271
	Lab	-	-0.373	-16.349	81.168	-1.145	-0.572	**0.839**	0.871	0.895	**-0.271**	-0.266	-0.260
	RLI	-	-0.587	-20.236	88.295	144.874	-0.487	0.827	0.857	0.876	-0.314	-0.303	-0.297
**Reciprocal Combination**	RGB	-	-0.296	-17.073	20.727	-93.151	124.971	0.824	0.872	0.880	-0.293	-0.287	-0.285
	HSI	-	13.726	-37.074	-15.731	5.904	0.295	0.828	0.863	0.863	-0.309	-0.296	-0.291
	Lab	-	2.661	-22.404	-4.844	-36.941	-367.321	**0.841**	0.870	0.902	**-0.279**	-0.253	-0.226
	RLI	-	-36.62	-26.815	59.015	-9.091	11.22	0.839	0.878	0.892	-0.293	-0.288	-0.281
**Power Function**	RGB	35.141	-2.121	1.362	0.032	-9.977	-23.123	0.838	0.862	0.904	-0.313	-0.265	-0.234
	HSI	-12.483	0.003	0.005	0.02	12.813	-24.621	0.812	0.864	0.869	-0.347	-0.334	-0.329
	Lab	57.563	-1.63	0.652	-0.398	-17.245	-24.276	0.829	0.867	0.904	**-0.234**	-0.184	-0.158
	RLI	-6.542	0.864	-0.442	-0.408	114.418	-18.779	**0.845**	0.885	0.890	-0.299	-0.281	-0.273
**Logarithmic Function**	RGB	-56.388	0.929	-0.456	-0.074	118.614	-18.458	**0.842**	0.860	0.900	**-0.288**	-0.266	-0.239
	HSI	-73.609	102.18	2.980	143.110	169.522	-19.865	0.813	0.854	0.866	-0.334	-0.321	-0.302
	Lab	62.805	23.162	47.639	-39.114	-15.354	-8.397	**0.842**	0.864	0.887	-0.306	-0.290	-0.284
	RLI	7.004	-78.473	-35.590	130.029	-13.058	39.253	0.829	0.861	0.868	-0.379	-0.359	-0.351
**Exponential Function**	RGB	84.911	-8.689	4.492	0.411	-9.396	25.619	0.847	0.884	0.912	**-0.274**	-0.235	-0.221
	HSI	89.014	-0.891	-0.072	0.411	-14.896	18.23	0.832	0.857	0.868	-0.424	-0.402	-0.394
	Lab	240.139	-4.471	-12.089	-3.518	-3.081	23.898	**0.848**	0.883	0.913	-0.303	-0.286	-0.274
	RLI	2.575	-12.728	10.724	4.674	-13.652	34.163	0.837	0.881	0.901	-0.399	-0.381	-0.374

*a*_0_~*a*_3_ and *b*_0~_*b*_1_ are model coefficients after adding the *GS*_*MCC*_ indicator

**Table 6:** Regression analysis results of error-in-variable models using color and *GS*_MCC_ indicators (*m* = 48).

Model	Color System	Coefficient						*R*^2^ (EIV)	$\bar{e}$ (EIV)	*R*^2^ (LSM)	$\bar{e}$ (LSM)
		*a*_0_	*a*_1_	*a*_2_	*a*_3_	*b*_0_	*b*_1_				
**Reciprocal Combination**	**Lab**	-	2.928	-21.938	-4.844	-34.029	-363.293	0.906	-0.219	0.902	-0.226
**Power function**	**RGB**	32.194	-2.485	1.879	0.187	-8.386	-24.565	0.915	-0.221	0.904	-0.234
	**Lab**	54.982	-1.419	0.592	-0.236	-19.284	-22.948	0.911	-0.151	0.904	-0.158
**Logarithmic function**	**RGB**	-53.643	0.987	-0.724	-0.184	115.845	-17.827	0.905	-0.231	0.900	-0.239
**Exponential function**	**RGB**	84.072	-8.428	4.938	0.917	-9.982	25.184	0.916	-0.212	0.912	-0.221
	**Lab**	237.374	-4.471	-11.927	-2.782	-4.274	26.248	0.935	-0.266	0.913	-0.274
	**RLI**	2.982	-13.084	11.524	3.194	-15.384	36.283	0.908	-0.312	0.901	-0.374

EIV: error-in-variable models; LSM: least square method models.

To verify the superiority of the error-in-variable model, we selected the models from Table 5 where *R*^2^ > 0.9 after adding *GS*_*MCC*_ and then used the error-in-variable model to perform the tests; those coefficients, *R*^2^ and $\bar{e}$ are shown in Table 6. Compared with the fitting degree obtained using the least squares method (columns 11–12 in Table 6), the error-in-variable model method improved *R*^2^ and reduced $\bar{e}$.

### Validation analysis of the total nitrogen content prediction model of sandalwood

Test validation samples were used to evaluate the models. The results are shown in Table 7. Compared to the least squares method, the error-in-variable model method both improved *R*^2^ and reduced the values of $\bar{e}$, *δ*^2^, and *MSE*, which demonstrates that the error-in-variable model method can obtain more accurate prediction results. As shown in Tables 6 and 7, the order of the model prediction accuracy of the modeling data and validation data is not exactly the same. For example, in Table 6, the Lab color system in the exponential function obtained the highest coefficient of determination. However, in Table 7, its $\bar{e}$ reached approximately 6—the second worst value in all of the models. To select the optimal model, we chose the *R*^2^ and $\bar{e}$ values obtained from the modeling data, and the *R*^2^, $\bar{e}$, *δ*^2^, and *MSE* values obtained from the validation data. Comparing the different models, the best result was “1”, and the worst was “7”. The results are shown in the last row of Table 7 and show that the optimal model is the exponential function using the Lab color system. The expression is as follows:

$$y=237.374e^{-(4.471\frac{L}{L\prime }+11.927\frac{a}{a\prime }+2.782\frac{b}{b\prime })}+26.248GS_{MCC}-4.274.$$

**Table 7:** Validation results for sandalwood total nitrogen content estimation models using both the least squares method and error-in-variable method (*n* = 24).

Parameter estimation method	Evaluation index	Reciprocal combination	Power function		Logarithmic function	Exponential function		
		Lab	RGB	Lab	RGB	RGB	Lab	RLI
**Least square method**	$\bar{e}$	-0.2209	-0.2181	-0.0938	-0.2149	-0.1991	-0.2632	-0.3514
	*δ*^2^	4.7656	4.2335	4.4567	4.7802	4.3777	3.7496	4.3438
	*MSE*	2.1942	2.0691	2.1132	2.1969	2.1017	1.9542	2.1136
	*R*^2^	0.8718	0.8901	0.8793	0.8719	0.8783	0.8894	0.8813
**error-in-variable model method**	$\bar{e}$	-0.2039	-0.1593	-0.0791	-0.1843	-0.1736	-0.2494	-0.3194
	*δ*^2^	4.2941	4.0193	4.3941	4.5932	4.0183	3.5931	4.0384
	*MSE*	2.0822	2.0111	2.0977	2.1511	2.0121	1.9119	2.0348
	*R*^2^	0.8956	0.8991	0.8957	0.8817	0.9063	0.9110	0.8931
	order	6	3	4	7	2	1	5

## Conclusions

With the development of “precision forestry,” there is a bright prospect for forestry information inversion and nutritional diagnosis acquired from digital image processing technology. The images used in this study were selected from different forest farms in the northern cities of Hainan Province. We used field servers to capture and monitor the health condition of sandalwood trees, and by using this equipment, a total nitrogen content prediction method was proposed.

Differing from previous studies, we defined a new indicator named *GS*, which includes two versions: *GS*_*MER*_ and *GS*_*MCC*_. The *GS* indicators together with the color factors are all treated as independent variables during the modeling process, and we used the error-in-variable model to estimate the parameters. This study developed a real-time and precise method to predict the total N content of sandalwood that meets the diagnostic requirement of automation. Our conclusions are as follows:

1. Sandalwood segmentation of field images can be realized by using the Lab color system. Due to its robustness to illumination changes and its large color range, the Lab color system provides a better result than RGB or other color systems. By applying the Otsu method to each channel, we found that the b channel is suitable for extracting green plants from the background, while the L channel is suitable for separating sandalwood from other plants. Therefore, this study combined those channels together with the Otsu method, median filtering, and morphological processing to complete the segmentation algorithm.
2. We propose a new indicator, named *GS*, which includes two versions, *GS*_*MER*_ and *GS*_*MCC*_, to describe the plant growth status. The combination of this indicator with the color factors provides more stable results regarding both accuracy and precision. After adding the *GS* indicators, the fitting degree was improved. We obtained better results when using *GS*_*MCC*_ than when using *GS*_*MER*_. Therefore, *GS*_*MCC*_, the minimum circumscribed circle, expresses growth status more accurately than does *GS*_*MER*_, the minimum enclosing rectangle.
3. Considering that errors exist in both the color and *GS*_*MCC*_ indicators, the error-in-variable model was adopted. Because of segmentation errors cause some color-value and pixel-number deviations, the traditional regression method is not appropriate. We found that the results obtained when using the error-in-variable method were better than those obtained using least squares estimation.
4. Five types of models are discussed in this study. Each model type was fit with four color systems: RGB, HSI, Lab and RLI. The optimal model of the total N prediction was selected by comparing the *R*^2^ and $\bar{e}$ values obtained from the modeling data and the *R*^2^, $\bar{e}$, *δ*^2^, and *MSE* values obtained from the validation data. The results showed that the exponential function using the Lab color system yields the most satisfying accuracy and precision in regression and validation.

## Supporting information

S1 Fig Images used for modeling. (TIF)

S2 Fig Images used for validation. (TIF)

S1 Table Soil nutrient content in the different study areas. (DOC)
