# Supplementary figures and images for: Preliminary research on total nitrogen content prediction of sandalwood using the error-in-variable models based on digital image processing
Source: PLoS One. 2018 Aug 21;13(8):e0202649. doi: 10.1371/journal.pone.0202649 (PMC6103514; doi:10.1371/journal.pone.0202649)

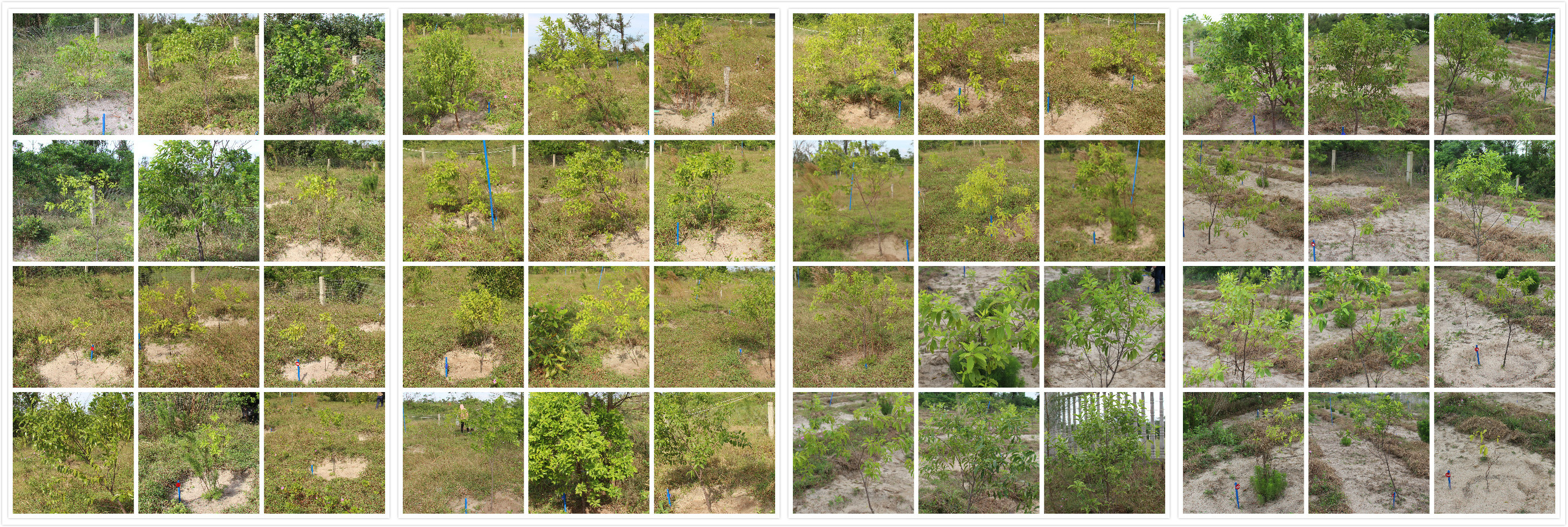

Supplement: S1 Fig — (TIF) [file pone.0202649.s001.tif]

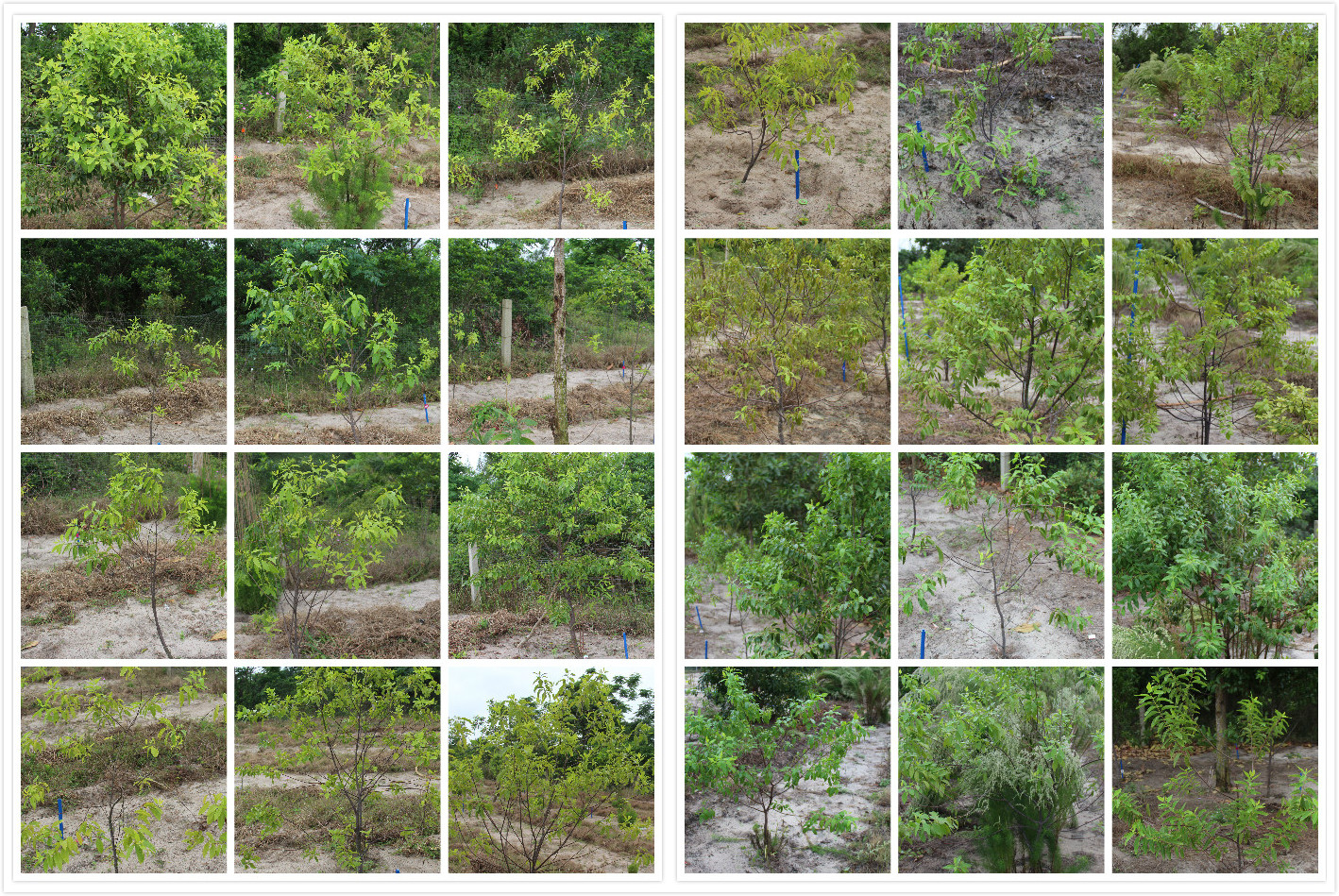

Supplement: S2 Fig — (TIF) [file pone.0202649.s002.tif]
