# Supplementary material for: Preliminary research on total nitrogen content prediction of sandalwood using the error-in-variable models based on digital image processing
Source: PLoS One. 2018 Aug 21;13(8):e0202649. doi: 10.1371/journal.pone.0202649 (PMC6103514; doi:10.1371/journal.pone.0202649)
Supplement: S1 Table — (DOC) [file pone.0202649.s003.doc]

**S1 Table. Nutrient content in soil in different study area**

| **Study area** | **Soil type** | **Nutrient content in soil before experiment** | | | | |
| --- | --- | --- | --- | --- | --- | --- |
|  |  | **pH** | **Organic matter content**  **(g/kg)** | **Total nitrogen content (g/kg)** | **Total phosphorus content(g/kg)** | **Total potassium content (g/kg)** |
| *Long hua district* | Yellow brown earth | 6.4 | 8.90 | 0.58 | 4.81 | 1.35 |
| *Qiong shan district* | Red earth | 5.0 | 0.53 | 0.79 | 1.39 | 1.27 |
| *Long lou town* | White sandy loam | 6.0 | 7.93 | 0.63 | 4.08 | 10.35 |
| *Wen jiao town* | Red sandy loam | 5.6 | 14.43 | 0.94 | 4.79 | 7.62 |
| *Ding an country seat* | Dark brown earth | 6.5 | 7.80 | 0.84 | 6.43 | 3.35 |
| *Tun chang country seat* | Latosol | 5.4 | 0.28 | 0.27 | 1.29 | 0.87 |
